# Taking Watsuji online: betweenness and expression in online spaces

**DOI:** 10.1007/s11007-021-09548-7

**Published:** 2021-04-17

**Authors:** Lucy Osler, Joel Krueger

**Affiliations:** 1 Center for Subjectivity Research, University of Copenhagen, Copenhagen, Denmark; 2 Department of Sociology, Philosophy, and Anthropology, University of Exeter, Exeter, UK

**Keywords:** Watsuji, Betweenness, Internet, Expression, Online space, Phenomenology

## Abstract

In this paper, we introduce the Japanese philosopher Tetsurō Watsuji’s phenomenology of *aidagara* (“betweenness”) and use his analysis in the contemporary context of online space. We argue that Watsuji develops a prescient analysis anticipating modern technologically-mediated forms of expression and engagement. More precisely, we show that instead of adopting a traditional phenomenological focus on face-to-face interaction, Watsuji argues that communication technologies—which now include Internet-enabled technologies and spaces—are expressive vehicles enabling new forms of emotional expression, shared experiences, and modes of betweenness that would be otherwise inaccessible. Using Watsuji’s phenomenological analysis, we argue that the Internet is not simply a sophisticated form of communication technology that expresses our subjective spatiality (although it is), but that it actually gives rise to new forms of subjective spatiality itself. We conclude with an exploration of how certain aspects of our online interconnections are hidden from lay users in ways that have significant political and ethical implications.

## Introduction

The Japanese philosopher Tetsurō Watsuji (1889–1960) was a broad-ranging and original thinker who developed important insights into cultural theory, ethics, religion, art, embodiment, and the self. He was also a skilled phenomenologist. His analysis of topics like intentionality, embodiment, space, and intersubjectivity not only predates insights developed by phenomenologists such as Sartre and Merleau-Ponty but can be used to extend their analysis in productive ways.

This paper has two main objectives: First, to introduce Watsuji’s phenomenology of *aidagara* (“betweenness”) and its analysis of embodiment, space, and intersubjectivity; second, to use *aidagara* to think through the dynamics of subjectivity and sociality within online spaces increasingly central to everyday life. We argue that *aidagara* develops a prescient analysis anticipating modern technologically-mediated forms of expression, connection, and engagement. More precisely, we show that instead of adopting a traditional phenomenological focus on face-to-face interaction, Watsuji argues that communication technologies—which now include Internet-enabled technologies and spaces—are expressive vehicles enabling new forms of emotional expression, shared experiences, and modes of betweenness that would be otherwise inaccessible. For Watsuji, these expressive vehicles and spaces aren’t mere add-ons to the self and its social capacities; rather, they are progressively incorporated *into* the self. Accordingly, they should be seen as constitutive parts of what he terms our “subjective spatiality”—that is, part of the embodied self and the rich pathways of “subjective extendedness” that establish enduring networks of interconnections with others.

We start with an exegetical analysis of *aidagara,* “subjective spatiality,” and “subjective extendedness.” Having provided some needed clarity to what these terms mean and how they relate, we then put Watsuji’s concepts to work in the contemporary context of online space. Using Watsuji’s phenomenological analysis, we argue that the Internet is not simply a sophisticated form of communication technology that expresses our subjective spatiality (although it is), but that it actually gives rise to new forms of subjective spatiality itself. We conclude with an exploration of how certain aspects of our online interconnections are hidden from lay users in ways that have significant political and ethical implications.

## Watsuji’s phenomenology of *aidagara*

*Aidagara* (“betweenness”) is a rich notion that does a lot of work for Watsuji. It is arguably the cornerstone of his phenomenology.^1^ Nearly everything he writes about ethics, social ontology, and the self in *Rinrigaku* (“Ethics”)—perhaps Watsuji’s most important work and one of only a few texts translated into English—in some way emerges from it.^2^ Clarifying this notion further is therefore crucial before applying it to subjectivity and online spaces.^3^

*Aidagara* is a common Japanese term that refers to relationships between people: being a sister, romantic partner, yoga teacher, doctor, or parent. But for Watsuji, the term has a more nuanced philosophical significance not fully captured by this ordinary meaning. In his work, *aidagara* is fundamentally an ontological category of human being and is not reducible to the ontic relationships of everyday life.^4^ So, while *aidagara* encompasses these ontic relationships and the roles that comprise them—it is impossible to exist in the world without inhabiting at least some of these personal and professional roles—the term is also meant to capture a more fundamental sense in which the very *being* of the subject is bound up with the rich interconnections it shares with others.^5^

For Watsuji, “betweenness” captures the interrelation between subjectivity, intersubjectivity, and space.^6^ We are embodied and situated subjects and, therefore, like other things in the world, we take up space. However, we do not merely take up space the way that tables, rocks, and trees do. We experientially *live* it. Lived space for Watsuji “is not so much the essential quality of a physical body as it is the manner in which a subject operates.”^7^ It is tied to our agency. And since “the manner in which a subject operates” is always shaped by the practices and spaces it shares with others, the lived space of betweenness is therefore an *intersubjective* space. These shared spaces have distinctive affective hues that play a central role in determining how we connect with the people and things around us. Watsuji’s phenomenological approach is, therefore, concerned with investigating the felt qualities of this spatiality and its constitutive relation with subjectivity and intersubjectivity. This experiential focus leads Watsuji to assert that the spatiality of betweenness “is not the same as space in the world of nature”; rather, it is “the betweenness itself of subjective human beings.”^8^

We will consider specific case studies of betweenness—both offline and online—throughout this discussion. For now, we can note that, for Watsuji, the spatiality of human betweenness takes many forms and degrees of intensity: from the bodily intimacy animating infant-caregiver interactions or sexual intercourse, to more general and expansive forms of betweenness within large groups, to the complex ways we organize the flow of information, communication, and transportation to open up or limit possibilities for social connection. This is what Watsuji seems to mean when he tells us that the spatiality of betweenness:

is not a form of intuition, but rather the manner in which multiple subjects are related to one another. It is not a uniform extendedness, but a dialectical one, in which relations such as “far and near, wide and narrow” are mutually transformed into one another. In a word, it is the betweenness itself of subjective human beings.^9^

The “dialectical” character of betweenness indicates that it is not something fixed or found pre-given in the world; nor is betweenness simply an a priori category of experience. Rather, it is a mode of being-in-relation-to-others that is actively *constructed*. By creatively engaging with environments that comprise our shared world, we determine what we do with the space of betweenness and how we connect with others in and through it.^10^ Observations such as these lead Watsuji to assert that: “I regard this subjective spatiality as the essential characteristic of human beings. Without it, the systematic relationships between personalities could not be understood.”^11^

The takeaway point—crucial for what follows—is that betweenness is something we play an active role in sustaining. It is not enough to consider space as though it is something we are passively *in* simply in virtue of our physical embodiment. Again, Watsuji insists that we must also investigate how the spaces we inhabit are transformed into something we *use*. Watsuji stresses this point because philosophy has, he argues, largely overlooked practical and experiential dimensions of space. Thinkers such as Descartes, Spinoza, Kant, and the German Idealists, to name a few, characterize space in impersonal Euclidean terms: “as a manner of the subject’s objectification or externalization”^12^—and, “[a]s a consequence, space has always been explored in connection with physical bodies or their motion and has never been investigated in connection with the activity of the human subject itself.”^13^ Watsuji’s phenomenology of *aidagara* addresses this oversight.

## Aidagara in action

We now consider examples of betweenness in action. They will help clarify two additional concepts central to Watsuji’s analysis: “subjective spatiality” (*shutaiteki kūkan*) and “subjective extendedness” (*shutaiteki na hirogari*). Watsuji’s discussion here is not always as clear as one might like. We will, therefore, attempt to bring these concepts into sharper relief before putting them to work.

As we’ve seen, “betweenness” is a broad concept that applies to the nature of the self and social world. It encompasses shared spaces in which individuals are located; the network of interpersonal connections that establish the affective character of these spaces; and, the way that modes of embodiment, consciousness, and the self are constituted by our ongoing interactions with others *within* these shared spaces. While “subjective spatiality” and “subjective extendedness” are aspects of betweenness, they are also more limited in scope.^14^

So, what are these concepts and how are they related to one another and betweenness more generally? “Subjective spatiality” highlights an idea introduced in the previous section: As embodied subjects, we don’t merely exist *in* space but also *live* it. Moreover, we use our lived spaces to fashion forms of betweenness within the I-Thou relationships of everyday life.^15^ “Subjective extendedness” has a slightly different focus. It can be found within the various ways we *express* our subjective spatiality.^16^ Activities of publication, communication, and transportation—some of Watsuji’s favorite examples—are processes that concretely extend our sense of subjective spatiality by opening up new forms of betweenness, new shared spaces and felt senses of possibilities for social connection. If “subjective spatiality” picks out a phenomenological feature of the embodied subject, then “subjective extendedness” refers to the artifacts, practices, and spaces we use to dynamically extend, enrich, and express this feature.

### Subjective spatiality in action

Let us turn to some examples. Consider one of Watsuji’s favorite cases of subjective spatiality in action: the bodily dynamics that regulate the character of infant-caregiver betweenness and support the development of early forms of self-consciousness and intersubjectivity. Infants are born with an implicit proprioceptive and kinesthetic sense of their own subjective spatiality. However, they have a limited ability to self-regulate their attention, emotions, and behavior. So, they depend upon the ongoing input of caregivers to bodily “scaffold” these limited capacities and assist them in realizing forms of self-regulation that would otherwise remain out of reach.^17^

For instance, in feeding^18^—one of the infant’s earliest experiences of betweenness—caregiver and infant form a dynamically coupled system via rhythmic cycles and back-and-forth interplay of short feeding bursts.^19^ Caregivers manipulate this shared space by using touch and gentle movements to prompt sucking responses and stabilize their fussy infants; infants, in turn, play a participatory role in shaping the character of this interaction by adapting and responding to these movements, which prompt new responses from their caregivers. Both transform the character of this betweenness through their subjective spatiality and realize a shared attentional and affective convergence.^20^ Watsuji observes that, within these early exchanges, “a [carer’s] body and [their] baby’s are somehow connected as though one. To contend that there is no such connection between them, because the link connecting them is not an actual cell is valid for physiological bodies but has nothing to do with subjective bodies.”^21^

These early examples of betweenness are developmentally significant. They provide shared spaces in which infants learn about themselves, including their body and its intersubjective capacities—i.e., their subjective spatiality—by helping to co-construct the spatial dynamics distinctive of these episodes of betweenness. For our purposes, it is important to note that the affective quality of these early interactions is crucial for drawing out and helping to refine these capacities. This becomes clear when we look at cases where the dynamics of early betweenness are disrupted.

Consider postpartum depression.^22^ Individuals with severe depression often exhibit disturbances of subjective spatiality.^23^ Among other things, they may report feeling a sluggishness or lack of embodied vitality; diminished affect and affective displays (e.g., facial expressions, etc.); and, a general loss of felt connection to the people, things, and spaces in their environment. Predictably, these disturbances impact how depressed individuals enact face-to-face betweenness. Clinically depressed caregivers often fail to consistently match or respond to the expressive displays of their infants.^24^ If the infant smiles or gestures, for example, the caregiver may respond with a flat or unenthusiastic facial expression, touch, or simply ignore their gaze entirely. Over time, the infant becomes increasingly attuned to the lack of vitality within these responses. They sense that something is affectively “off,” that their expectations have been thwarted and a violation of interactive norms has occurred. So, they use various strategies to re-engage their caregiver’s attention and restore the reciprocity of the interaction. However, if the caregiver consistently responds to these strategies by avoiding the infant’s gaze or by failing to match the intensity, focus, or timing of their expressions, the infant will become increasingly restless and distressed; they instead turn to self-soothing strategies (e.g., rubbing their own hands or head) in order to receive affective feedback missing from their caregiver’s diminished subjective spatiality.

These early disruptions of betweenness not only have a short-term impact on the infant’s affect and behavior; they potentially have long-term consequences, too, and may lead to maladaptive strategies for regulating and sharing emotions later in life.^25^ The point of these examples—and the reason Watsuji returns to them on multiple occasions—is to highlight how betweenness consists of more than mere physical proximity. Again, it is something we actively create and sustain—a way of connecting directly with others that involves, among other things, a synchronization of the movements and bodily expressions that are part of our subjective spatiality. For Watsuji, the dynamics of subjective spatiality are the foundation of our I-Thou relationships. To connect with others is thus not primarily a meeting of the minds (i.e., an exercise of our folk psychological capacities), but rather a more fundamental sharing of embodied (inter-)subjective spatiality and lived space. This is what Watsuji seems to have in mind when he writes: “[W]hen *I* as the subject of practice stands face to face with *Thou*, *Thou* stands face to face with *I* as the subject of practice. One’s physical body exhibits personality in every part and, hence, lures another’s personality in its every motion.”^26^

### Subjective extendedness in action

The dynamics of subjective spatiality described above are not confined to early infancy. They are enacted in various ways throughout our lives, within our embodied engagements with others. Although the previous examples focused on face-to-face I-Thou interactions, engagements with the *material environment* also regulate and sustain these dynamics and the forms of betweenness they generate. “Subjective extendedness” helps to think through this dimension of betweenness-construction.

Watsuji argues that cultural artifacts and practices are ways of both expressing our subjective spatiality and managing betweenness. For example, ordinary items encountered in everyday life “are neither simply object-like nor economic concepts. Rather, they are something which, as clothing, food, and shelter, expresses these respective moments […] of human existence.”^27^ Different styles of clothing—“ceremonial clothing, visiting clothes, daily clothes, uniforms, children’s clothes, baby clothes, and so on”—express subjective spatiality insofar as they bear traces of the different activities and forms of life (rituals, parenting, work, play, etc.) that give these styles their distinctive meaning. Watsuji concludes that “there are thus no goods in which *ningen sonzai* [human existence] is not expressed.”^28^

But things are not that simple. Part of the challenge of interpreting “subjective extendedness” comes from recognizing that Watsuji also sometimes has a different—and *stronger*—sense of “expression” in mind.^29^ What we have just considered is a *weak* sense of expression, in that cultural artifacts like clothing indirectly refer to (express) dimensions of human existence that give them their intelligibility. However, whereas clothing can be said to express human existence in that it bears traces of our activities and forms of life, other artifacts and spaces express human *being*—and not just (ontic) human *existence*—in that they are, as Johnson puts it, “the self-externalization of human beings, that is, of us ourselves (*soto ni deta wareware jishin de aru*).”^30^ In other words, these artifacts and spaces enlarge and enrich our subjective spatiality by *bringing new forms of subjective spatiality and betweenness into being*. We use them to construct distinctive relationships of betweenness with one another—each with a unique intensity and character—and to animate forms of shared feeling that sustain these relationships across distances large and small. For Watsuji, subjective extendedness “arises because human beings, despite dividing themselves into a great number of subjects, nevertheless, strive to constitute a connection among themselves” by enlarging the relational spaces that sustain such connections.^31^ This extendedness, Watsuji tells us further, “is a ‘tension' within the interconnection of the acts of subjects, which changes its strength and degree of inclusiveness in accordance with the multiplying and unifying of subjects.”^32^

The “tension” Watsuji refers to here is specified, in part, by the character of the spaces our material resources help us construct. Some of these resources create relatively open and accessible spaces in which large numbers of subjects can easily come together and connect; others are more exclusive, intimate, or have a higher bar of entry and, therefore, animate a different relational character and style of interaction. Our sense of subjective extendedness may, therefore, expand or contract as we move through and negotiate these different spaces. A key point to remember, however, is that since Watsuji is concerned with *lived* space (i.e., subjective spatiality), the “strength and degree of inclusiveness” of a given relational space—and the degree to which it extends our sense of subjective spatiality—is not tied to its *quantitative* size. One can feel deeply alone and excluded from a physically large space such as a university or city; or, a felt sense of exclusion may arise as one attempts to negotiate unwelcoming spaces that are not set up to accommodate non-white bodies,^33^ say, disabilities,^34^ or autistic styles of movement and social interaction that depart from neurotypical expectations.^35^ This exclusion may lead to a felt *contraction* of subjective spatiality commensurate with one’s inability to comfortably settle into these spaces. Conversely, one may feel deeply and intimately connected to others—and, thus, experience an intense *extension* of subjective spatiality—in quantitatively small spaces such as the apartment of a close friend, a cozy restaurant, or even in some online spaces.

Before turning to these online spaces, let us briefly consider a few more examples of subjective extendedness. Consider the material structure of a Catholic confessional booth. It is deliberately crafted to manage spatial and affective dynamics of religious betweenness.^36^ Its design—normally a wooden booth with a central compartment covered by a door or curtain—shields the penitent from the gaze of others, including the priest who can only hear the confession spoken through a lattice dividing the compartment in two. Its structure is designed to minimize embarrassment and distraction by occluding the outside world. Instead, the penitent is encouraged to feel a sense of intimacy, privacy, and trust within this space, a feeling that compels them to speak freely about their sins and seek forgiveness. The physical structure and space of this artifact, thus, shapes the attention and affect of priest and penitent; both speak in quiet tones, and the confined space limits unnecessary movements and distractions. But this structure also shapes the *lived* space of the booth, its affective hue and distinct styles of interacting and intensities of betweenness that reflect this affective hue. Again, it animates a felt sense of openness, vulnerability, and trust that might not be achievable were the practice to unfold in a more exposed setting. So, although the physical space of the confessional booth is quite small, it nevertheless can be said to *extend* an individual’s sense of subjective spatiality by establishing a relational space that offers felt possibilities for an intimacy and intensity of shared connection unavailable in other (perhaps physically larger) spaces of betweenness.

Communication and transportation technologies are another of Watsuji’s favored examples. These technologies (e.g., cell towers, broadband lines, roads, train lines, etc.) clearly have physical properties that extend through space. “This extendedness seems at first sight to be taken as physical space,” Watsuji tells us — “but this is not the case.”^37^ The felt significance of a road, for example, “has nothing to do with that physical thing that is of a certain width and length in merely physical space.”^38^ Rather, communication and transportation technologies are experienced not merely as physical objects but as “an expression of human connection.”^39^ This is because these technologies extend our sense of subjective spatiality by creating new opportunities for betweenness. They do so by opening up new and more immediate possibilities for connection and shared experience not bound by physical space or by the physical properties of the technologies that extend through physical space.

To return to our earlier distinction: For Watsuji, these technologies do not merely express human existence in the weak sense of “expression” considered above, in that they indirectly refer to the agents responsible for their creation or the forms of life in which they are embedded (although they clearly do). Rather, they are also expressive in a strong sense, that is, expressive of human *being*. Again, this is because these technologies enlarge and enrich our subjective spatiality by bringing new forms and intensities of betweenness into being that would not otherwise be available. For example, “the intensity of social connections is given expression to by the intensity of railway lines, as well as by the frequency of trains.”^40^ Greater railway lines and more frequent trains generate a stronger and more enduring sense of connectedness to the outside world. They contract physical distances and thereby open up a felt sense of possible spaces and pathways of connection that *span* these contracted distances—an extension of subjective spatiality that would not occur without their presence.

Communication technologies also accomplish this subjective extension but even, perhaps, in a more intense manner. This is because they not only *contract* but experientially *eliminate* these distances. Watsuji observes that as communication technologies advance “we will be emancipated from the restrictions of distance and will be able to participate in any conversation as freely as we wish.”^41^ This felt emancipation from the constraints of our local physicality is an extension of our subjective spatiality. Wherever their location in the world, others are felt to be accessible for connection and sharing simply by writing a letter, picking up the phone, or opening an app on our smartphone.

Of course, as a sensitive phenomenologist, Watsuji is attuned to the experiential manner in which these “spatial connections vary in accordance with the various ways of communication.”^42^ A key feature of this variation concerns the *temporality* of our exchanges. As we considered above, our face-to-face I-Thou interactions are ways of connecting directly with others in shared spaces that involve, among other things, a real-time coordination of movements and bodily expressions that are part of our subjective spatiality. As we develop, language also becomes a central part of these exchanges. Within I-Thou interactions, we expect that our gestures and utterances will elicit immediate responses from others (and vice-versa). This shared sense of temporality is part of what gives our I-Thou interactions their intensity and affective character and allows us to share experiences as readily as we do.

However, “[i]t is obvious,” Watsuji continues, “that a direct conversation and the use of a messenger as a mediator constitute practical connections quite different from one another.”^43^ When we write a letter, for instance, we do not expect an immediate response. The “local restriction inherent in [this] social connection” remains in that we are sensitive to the time it takes for our letter to reach its recipient. Moreover, “[i]f we receive a response at a time when we have almost detached ourselves from the state of mind we were in while writing the letter, then we are unlikely to share the same state of mind” either with ourselves or the recipient, which will further diminish the affective intensity of this exchange.^44^ Our practical expectations, thus, play a role in shaping the character of the relational spaces our letter-writing practices open up and the sense of interactive possibilities we feel within these spaces. However, when we know that we can quickly tap a smartphone app and immediately engage in a real-time video chat with a faraway friend, a felt sense of this “local restriction…will be likely to disappear.”^45^ The affective hues of our online chat spaces, thus, differ from the relational spaces of letter writing. They are specified by our practical expectations about the real-time and reciprocal possibilities presented by this mode of interaction, and, therefore, generate a distinctive style and intensity of betweenness.

The key takeaway point is this: For Watsuji, the heterogeneity of our betweenness experiences—including their affective character—covaries with the artifacts and spaces that generate these experiences. These artifacts and spaces enlarge and enrich our subjective spatiality by bringing new forms of subjective spatiality and betweenness into being. We use them to create distinctive relationships and forms of connection with one another—each with a unique affective intensity and character—and to animate forms of shared feeling that sustain these connections across distances both large and small. This is what Watsuji means when he says that interaction with the material world helps us enact forms of “subjective extendedness.”

## Online social space

We have discussed how Watsuji not only conceives of embodied subjects as essentially spatial and our interpersonal encounters as taking place *between* individuals, but also his argument that “subjective spatiality” can be concretely extended into the material world as “subjective extendedness.” We now put Watsuji’s notions of “subjective spatiality” and “subjective extendedness” to work in the realm of online space(s). We start by considering how the Internet looks similar to other mediums of communication that Watsuji discusses, such as letters and telephone calls, and as such may be considered an expression of human interconnectedness—a form of subjective extendedness. However, we go on to make a stronger claim: Rather than just being a means of communication, the interactive nature of our interpersonal encounters online can be conceived of in terms of subjective spatiality proper. We show how Watsuji’s notion of subjective spatiality can help us unpack what we mean when we talk about “online space.” We argue that while online space is not a geometric Euclidean space, it is a *lived social space* that fits with, and can be helpfully elucidated by, Watsuji’s notion of subjective spatiality.

### Online space and betweenness

Despite its interest in sociality, phenomenology has said very little about interpersonal encounters online.^46^ Phenomenology of sociality typically focuses on embodied face-to-face encounters. Online interactions seemingly take place in a virtual *disembodied* arena and, therefore, might seem to lack the concrete qualities that phenomenology has taken most interest in. A similar lacuna is found in work on the Internet within embodied and situated approaches to cognition, which assume that details of our body and world do not matter much when it comes to understanding online interactions. These approaches primarily focus on the *informational* nature of the Internet and how online spaces augment our information-processing capacities and memory.^47^ However, within these quarters there is increasing recognition that the Internet is not simply an informational resource but also a highly *social* space—“a space in which people are able to interact, socialize and share information” and emotions.^48^ This recognition calls for reassessing the way details of our embodiment and spatiality potentially enter into and shape our online encounters with others.

What does it mean, then, to say that the Internet is a social *space*? When we “go online” we do not enter into online space the way that we enter into a cafe, a house, or a lecture hall. When we talk of online space, we are clearly not referring to a *physical* space. It makes no sense to say that WhatsApp is to the left of Facebook; we cannot plot the location of Instagram on a map or with coordinates, or move to a different position to get a better view of a specific tweet or an element of Twitter’s user interface. So, when we talk about online spaces, are we simply being metaphorical? Or do our online experiences have spatial dimensions? If so, how should we understand these dimensions and how they shape our experience of sharing online spaces with others?

This is where Watsuji can help. As we’ve seen, the betweenness that defines our spatial relations with others—I-Thou relationships of everyday life—is not fundamentally Euclidean geometric space but rather *lived* space, the subjective spatiality of lived bodies. Watsuji argues that to exist as an embodied human being is to exist in—and *as*—betweenness. We argue that this is just as much the case when we consider our online encounters as it is our offline ones.

Recall that for Watsuji, transportation and communication technologies are “expressive” of human being in a strong (i.e., *ontological* and not merely *ontic*) sense. They enlarge and enrich our subjective spatiality by bringing new forms and intensities of betweenness into being; these material resources contract physical distances by opening up a felt sense of connectedness and interactive possibilities that spans these distances. Interacting with these resources is, thus, a way to enact forms of “subjective extendedness” bringing individuals into lived contact with one another even when they do not share the same physical space.

We might, then, suppose that the Internet facilitates betweenness by serving as a mediator for communication akin to letters or telephones. However, we argue that we do not only use the Internet as a sophisticated tool for *effacing* space by communicating with those who are physically distant from us. We also experience it as a resource for *creating* space; online spaces that we experientially share *with* others. As Watsuji indicates, when our communications with one another are sped up to a sufficient level, we are no longer communicating with others in a mediated or indirect way but are instead in “direct conversation” with them. Indeed, in this prescient paragraph, Watsuji seems to precisely anticipate the rise of something like the Internet:

It is obvious that a direct conversation and the use of a messenger as a mediator constitute practical connections quite different from each other...Under circumstances in which a response to a letter is delivered after a month’s interval, we cannot be said to be engaging in a conversation in an active manner. If we receive a response at a time when we have almost detached ourselves from the state of mind we were in while writing the letter, then we are unlikely to share the same state of mind. On the other hand, if and when postal services spare no time in delivering words from one person to another both quickly and frequently, then we shall be able to share pleasures as well as pains. A community of being would thus be realized.^49^

What Watsuji emphasizes here is that direct conversation not only takes place in the context of the temporal dynamics distinctive of our face-to-face interactions; it can also arise when there is *active and reciprocal interaction* between participants in other communicative contexts, too. When this sort of active conversation occurs, then a “community of being”—a new form of betweenness—can emerge.

We think that the activity available on the Internet can put us in direct conversation with one another, allowing for active engagement that is not just a subjective extension *indirectly* expressing the connectedness of individuals (i.e., the way the presence of a letter on a desk indicates an author) but which can properly be considered a form of subjective spatiality. What is unique about the Internet, though, is that the online *preservation* of our conversations that we engage in—e.g., threads archived on WhatsApp chats, Twitter, and blog comments etc.—can also be considered technological artifacts that express our spatial extendedness. Thus, the Internet allows for spatial subjectivity akin to that which we find in I-Thou face-to-face interactions, as well as existing as a more enduring form of spatial extendedness. We will discuss these two different forms of betweenness on the Internet in turn.

### Subjective spatiality and spatial extendedness online

Like our offline worlds, our online worlds are spaces of *activity*. There are a multitude of things we can do on the Internet: browse websites; message friends; upload photos; scroll through people’s timelines on Instagram, TikTok, or Facebook; have a Zoom meeting with colleagues; edit documents in real time with our co-authors in Google Drive; play D&D on roll20; watch a video on YouTube and peruse the comments; and so on. Across these examples, we are engaging with others—either *explicitly* (e.g. when messaging or video calling) or *implicitly* (e.g. reading other’s blogs and comments).

One might be concerned here that although there are numerous ways of seeming to engage with others online, we are not truly in relation to them as we are not embodied agents *in* online spaces. In other words, one might think that we only ever engage with the *signs* and *symbols* of others online (e.g., their video image, texts, etc.), and not their subjective spatiality proper. Fuchs, for example, is skeptical about whether we really encounter the other online at all.^50^ He argues that online communication is, necessarily, a *disembodied* form of communication: “what is in fact lacking is interaffectivity, i.e. the direct feedback from the embodied contact, based on emotional cues and expressive gestures.”^51^ The risk of disembodied online communication, Fuchs continues, is that rather than encountering the other in their full subjective spatiality, we are instead prone to *projecting* our own emotions, our own imaginative interpretations, onto them. This suggests that even though we might think we are encountering the other in an authentic I-Thou mode, we instead only have an experience “as-if” we were encountering the other.^52^ In other words, we are not achieving direct contact with them, only an indirect or mediated representation. This certainly seems to be a bleak outlook, if true.

We disagree with this prognosis.^53^ Once more, Watsuji’s phenomenology of betweenness can help see why these worries are unfounded. Recall that Watsuji outlines the I-Thou relationship as follows:

...when I as the subject of practice stands face to face with Thou, Thou stands face to face with I as the subject of practice. One's physical body exhibits personality in every part and, hence, lures another's personality in its every motion.^54^

As we’ve seen, this I-Thou relationship is dialectical. Our experience of the other arises out of the back-and-forth embodied and interaffective dynamics that animate our engagements. This characterization of the I-Thou relationship, at least initially, might seem to support what Fuchs claims in terms of requiring us to be physically together for such a relationship to emerge. However, Watsuji’s phenomenological analysis offers further nuance that resists this quick conclusion. He goes on to state that: “gestures or expressions of *Thou*, who stands over against *I*, are neither motions of physical bodies nor mere vibrations of air but are the relationship of a subjective *Thou* with an *I.*”^55^

When we interact with each other, we are not merely physical bodies making noises and movements at one another. Rather, we are living subjects—*subjective spatialitie*s—interacting with and co-regulating one another in an active, expressive manner. Given this analysis, it seems that we can liberate the I-Thou relationship from the proximal constraints of face-to-face interactions. As noted above, Watsuji anticipates exactly this when he imagines the postal service speeding up our communicative exchanges to the point where our conversation is no longer *mediated* but becomes a form of direct, active, and reciprocal engagement—in other words, a means of realizing the dynamic qualities needed to bring subjects into direct contact with one another.

*Contra* Fuchs, then, we argue that interaffectivity and expressivity can, and indeed does, happen in our online interactions. To illustrate this, we take WhatsApp chats as our example. On WhatsApp, we can send instant messages to one another in real time; see when the other is online, typing, or last active; see when our messages have been delivered and read, or perhaps ignored. Note that while many of these features are not unique to WhatsApp, we want to be careful not to suggest that all our online interactions are the same; the Internet is not one homogenous space but a collection of various platforms, each with different forms and possible styles of interpersonal interaction available.^56^

When we send instant messages to one another, we tend to adopt a style of communication that reflects our face-to-face conversations. We typically do not use full sentences; we respect turn-taking rhythms; and conversation flows dynamically forward.^57^ In other words, we are keenly *responsive* to one another, sensitive to each other’s tone, style, and norm-governed rhythms. That this is the case is highlighted when we reflect upon how our conversation style is different online when we are interacting with different people (e.g., friends versus professional acquaintances). We are not only sensitive to the style and tone of others. This sensitivity, in turn, affects our *own* tone and style. Conversation—both offline and online—is, therefore, not merely the informational aggregate of what the two of us are saying. It is a quality of relatedness—*betweenness*—that emerges between us. It is active and dynamic; it *unfurls*.

What we want to emphasize here is that when we are instant messaging with another on WhatsApp, we are not simply sending written words to one another. These words are expressive; they have a certain tone, dynamism, and qualitative style. And what arises in these exchanges, then, is not just an (indirect) expression of subjectivity but an active, unfolding I-Thou interaction—an instance of subjective spatiality. Our WhatsApp conversations can regulate, scaffold, and influence our dynamic interaction with one another. Moreover, these online social spaces we find ourselves in with others have a particular affective hue to them, a distinctive atmosphere that gives them their felt character and sense of interactive possibilities Osler (2021b). When we are excitedly chatting with our friends, for example, the space of that exchange is colored by a betweenness of comfort, ease, and happiness. However, if we are arguing with someone over WhatsApp, we experience that space as tense, uneasy, and uncomfortable. This affective character is established by bursts of terse and sharply-worded text, followed by the awkwardness of seeing a typing indicator that goes on for too long, showing that the other is typing and retyping their response, unsure of how to respond. This expressive gesture intensifies the awkwardness and discomfort permeating the interaction.

The rhythms, style, and tone of our interactions are dynamic qualities that shape and regulate the sense of betweenness we experience. What we contend is that our online interactions, even though predominantly articulated through text, also modulate the betweenness of the participants and can, therefore, be considered part of our subjective spatiality. Just as Watsuji highlights that the physical body can express the other’s personality so, too, can one’s online communication. These observations shed light on why we do not experience all our online interactions as being the same, as having the same expressivity or felt character. They are distinct because we are mutually expressive *in and through* our unfolding online conversations. This is a shared, reciprocal process; we are sensitive to one another, affect one other, and play an active role in drawing out and regulating each other’s experiences within these online spaces.

As such, we think it wrong to see our online interactions as expressive of the other merely in terms of subjective extendedness. Our conversations are not simply an expression of human spatiality in the material world, the way roads and telephone lines are, but are instead instances of subjective spatiality. If we understand such online interactions as instances of subjective spatiality, this helps us understand the Internet as a social space, for it is another instantiation of subjective spatiality at play.

What, though, is unusual about the Internet is that unlike our face-to-face interactions, our direct, active conversations online are often preserved. As such, not only does betweenness exist online in terms of the subjective spatiality of our direct conversations, but these conversations are concretized in the material world. The point we are making here is that in online space we find examples both of subjective spatiality and spatial extendedness (sometimes in relation to the same conversations but at different times). Where interactions are no longer dynamic, interactive, flowing conversations, we no longer find the spatial subjectivity of the *I-Thou* relationship. However, what we do still find are expressions of human interconnection, a form of spatial extendedness.

For instance, we can look back at our old threads as evidence of our connectedness to others on the other side of the world, even though we are not in active dialogue with them at that moment. Personal threads in WhatsApp or chains of comments on news sites, blogs, Facebook or YouTube persist over time. They are there even after the initial interaction, which might have constituted the subjective spatiality of an I-Thou relationship, has passed. These are preserved forms of interconnectedness between individuals, which are expressive of human relatedness. We might also think of how systems such as “liking” found on Facebook or Instagram are forms of spatial extendedness. Here, human beings are interacting with one another, connecting with each other. However, the interactive richness required for an I-Thou relationship is likely missing.

Betweenness in the form of spatial extendedness, then, can also be found online. Like the other forms of spatial extendedness that Watsuji describes, such as letter boxes, train lines and message boards, these do not only express human connectedness but also the intensity of human connectedness. Watsuji describes how the number of train lines leading out of a city expresses the intensity of that city’s interconnectedness with other communities. Analogously, the number of likes and comments that an Instagram photo receives expresses the connectedness of the post. We even experience this in an affective manner. A post with very few likes, for instance, is experienced as somehow “emptier” than a post with a lot of engagement.^58^ This is nicely captured by Donath and Boyd’s term “public displays of connection.”^59^ This expressive connectedness is, then, still present on the Internet.

## The hidden nervous system of the internet

So far, we have discussed how Watsuji can help us unpack what it means for us to speak of the Internet as an online *space*, and in particular a social space. We have argued that Watsuji’s phenomenological analysis of betweenness, subjective spatiality, and subjective extendedness, can be fruitfully applied to the online realm as well as the offline one. Our approach takes seriously the expressive, communicative, and interaffective aspects of our online interpersonal encounters and social space without falling into reductive conceptions of the Internet either as simply an information source or as being a place of cold, symbolic, and indirect interaction. This Watsuji-inspired approach allows us to do justice to the rich affective-expressive experience of online interactions, online communities, interpersonal relations, and forms of connectedness online spaces offer.

However, we now want to enrich this understanding of the Internet in terms of betweenness by considering an important *difference* between the online realm compared to the offline one.^60^ As highlighted above, Watsuji discusses how visual markers of communication and connection, such as train lines, roads and letter boxes, are forms of subjective extendedness that *express how communities are connected to one another*. A road, for example, expresses the connection of one town to the next and opens up felt possibilities for mutual interaction between the inhabitants of these towns. Moreover, the state of the road, how well-worn it is, its position (e.g. a main road rather than a small path), expresses not only the connection but the practical and experiential “density” of the connection. Physical characteristics of the road modulate the affective character of the betweenness experience it generates.

Online, however, our communities are not, at least in principle, bounded by geography.^61^ You can flit between WhatsApp groups with individuals from all around the globe; you can read the New York Times while sitting in your home in Copenhagen; you can coordinate Netflix Party watch-a-longs with anyone with access to the Internet. In many ways, our communities are all interconnected instantaneously, without a need to travel along paths, roads, or train tracks. We might think, therefore, that the markers that visually express our community interconnections that Watsuji describes fall away when we enter the online world. We no longer exist in geographically demarcated villages, towns, and cities but are part of a globalized network. Indeed, Watsuji presciently captures this when he states that: “If this method of communication advances to its fullest extent, then we will be emancipated from the restrictions of distance and will be able to participate in any conversation as freely as we wish.”^62^ On the Internet, our spoken words are no longer “hampered in achieving its objective because of the long distance involved.”^63^ Online spaces open up interactive possibilities not bound by physical space or by the physical properties of the technologies that extend through physical space. Online we are as close to our neighbors in Copenhagen as we are our friends in Exeter.

The communities that we engage with online are, at least usually, not delimited by one’s geographical setting but spring up based upon personal connection or around shared objects of interest. We can create communities online that center around shared interests (e.g. books, politics, and art) or shared activities (e.g. playing Dungeons and Dragons online, watching movies together).^64^ Although the Internet allows for a robust experience of betweenness through online activity, it might not give rise to “an expression of human connection” in the same way that a road does. It is not immediately clear how we see the Internet bringing different *communities* together. Perhaps this is simply because the Internet eliminates the local restrictions Watsuji spoke of, leaving us in an entirely globalized society.

This conclusion is somewhat naïve, however. Although we are freed from geographical limitations, we do not have one “online” community but multitudes of communities that, in many ways, are still separated from one another. Think, for example, how the rise of the Internet has resulted in more explicit recognition of the echo chambers that arise online.^65^ The communities that we habitually engage in are demarcated by our common interests, our politics, our culture, our taste. These online echo chambers have few visual markers that connect them to other communities. When someone sits down to read the Guardian on their screen in the morning, there are few expressive spatial connections that show how the articles, the contents, and the reading community connects with other news-reading sites, particularly those of a different political leaning or agenda. Even if a Breitbart reader, say, were to stray onto the Guardian website, unless they leave an explicit comment, their presence in this space would go under the radar for most lay-users, leaving no visual path behind them.

This, though, is not to say that the communicative, connective, and expressive “nervous system” that Watsuji describes is not on the Internet at all. Those with the right access and the right skill can track the well-travelled roads based on site hits and IP addresses. This kind of information directly parallels the nervous system that Watsuji cashes out in terms of travel and communicative connections.^66^ That this is the case is even reflected in our language when we discuss how much “traffic” a site, an article, or a post gets. Indeed, some of this “traffic” *is* visually available to users. As noted above, on Instagram, for instance, the likes and comments that a post gets is an expressive, visual form of spatial extendedness that resembles Watsuji’s train lines. It indicates the intensity of betweenness that has arisen between the individual who posted and the viewers moved to like or comment. However, what might be missing is the broader intricacy of the “nervous system” of human interconnectedness that we see in the offline world. Online we cannot, with such ease, see where people have come from, which communities are interacting with one another, where the “roads” are. Watsuji describes how an “intersecting point of arteries of traffic expresses intense human social intercourse.”^67^ While we can see the bunching and coagulation of interaction online, the trails or roads are harder to find and trace.

What is available to the user online is more obscure, more limited than offline. In our offline worlds, it would be difficult to hide the number of roads, train lines, and post boxes that a town has, difficult to mask this interconnectedness of the community beyond its immediate geographical setting. Online, though, large parts of the nervous system sit behind the veil of the platforms we engage in. When we go on Instagram and Facebook, the lay user does not get exposed to the full range of Internet traffic information that exists. While Jeff Bezos and Mark Zuckerberg might have a whole raft of information about how users and communities are connecting with one another, other sites, and products, the common user does not. There is, then, a sense in which our everyday expressive subjective extendedness is *partially masked* when we go online.

This partial masking of the nervous system of the Internet has political implications. The betweenness that exists online can end up coalescing, bunching, and pooling in certain ways that is not immediately obvious to the common user. We might describe the Internet as having a kind of “islanding” effect, where communities become oddly segregated and cut off from one another. In theory, when we go online, we have access to the whole Internet community, living in a global betweenness. In practice, though, we still tend to operate in bubbled communities, online villages and towns that are often bounded not by trees, lakes, mountains, and seas but by politics, opinions, tastes, language, and interests. Indeed, personal algorithms on social media platforms routinely exacerbate this bubbling effect by promoting content to us that fits our interests and limiting our exposure to other communities.^68^ If we cannot see the roads that lead us out of our village, we can come to think of our village as *the* community. One might not think that this is particularly unusual. In our offline worlds, this segregation happens due to geographical formations and distances. Someone sitting in Exeter, say, is cut off from communities in Madagascar. However, what is different, and potentially malevolent, about the Internet is that these online “islands” might exist in such a way that the users do not realize that they are on an island. Where the expression of human interconnection is masked, we risk forgetting that this broader interconnection is there at all, or that it is something we might miss or ought to seek out.

If the visual markers of connection between online communities are missing, what is also missing are felt possibilities for mutual interaction. This could lead to online communities becoming increasingly entrenched and increasingly solipsistic, further enforcing the echo chamber effect. Making connections between these online communities may therefore be experienced as more difficult and more perilous, with each individual who makes the attempt feeling as though they are charting a new path.^69^

## Conclusion

Drawing upon Watsuji’s phenomenology of *aidagara*, we have argued that his analysis of betweenness, subjective spatiality, and subjective extendedness can be fruitfully applied to the online realm. Rather than conceiving of the Internet simply as an information resource or a message delivery system, our approach takes seriously the expressive, communicative, and interaffective aspects of our online interpersonal encounters and social space. Watsuji’s intricate analysis of human connectedness and subjective spatiality provides a helpful framework for exploring the complexity of our online spaces and social encounters, allowing us to move beyond simply seeing our online interactions as mere markers of betweenness and to instead properly account for the affective richness of *being* with others in online spaces.

Having defended a robust notion of betweenness online, emphasizing how our online interactions can be constitutive of subjective spatiality proper, we have tempered our account by turning to ways in which betweenness is also constrained online. While the Internet, at least in theory, opens us up to global betweenness, in day-to-day life we still exist in bounded communities online, even in bubbles or echo chambers. Due to the structure of online space, much of the nervous system of the Internet and the connections between online communities are hidden from the lay-user. As such, many of the expressions of interconnection between online communities are lost. We have suggested that this can result in not only the creation of echo chambers but also in the entrenchment of them. Where we do not see paths leading out of our online communities, we do not feel the possibility for connection with those who fall outside our everyday networks. Consequently, while betweenness can and does take place online, it does not take place equally between all users and, as we’ve noted above, can be manipulated by those who design the online spaces and platforms that we use. We would also be remiss if we fail to note that we do not live in a world where there is equal access to the Internet, as well as recognize that Internet access itself has its roots in politics, often relying on national infrastructure and government policies. In recognizing that online spaces open up opportunities for betweenness, we also must recognize that there are political and ethical consequences to this. With its overtly ethical orientation, Watsuji’s phenomenology of *aidagara* offers rich theoretical resources for thinking through some of these consequences.

While we have focused here on providing an initial account of how subjective spatiality is enacted in the online sphere, what needs further exploration is how betweenness online develops and how different styles materialize between individuals. As the infant learns how to interact with their caregiver, so must we learn to interact with others online in an expressive, interaffective manner. Likewise, the affective contour of betweenness varies depending upon the style of an interaction. To enrich our account further would involve exploring in detail how we skillfully learn to interact with others online, as well as examining how different contours of betweenness arise. Finally, given that Watsuji understands *aidagara* as constitutive of the self, an additional line of analyses suggests itself: If we increasingly enact our spatial subjectivity in an online forum, what (if any) implications might this have for the constitution of the self more generally?

## Data Availability

Not applicable.

## Footnotes

1 McCarthy (2011a), Krueger (2013a), Johnson (2019).

2 Shields (2009).

3 We will primarily draw upon the discussion of betweenness found in Chapter 9 of *Rinrigaku*, “The Spatiality of a Human Being.”

4 Johnson (2019, p. 84).

5 McCarthy (2011b), Krueger (2019). Watsuji’s approach to the self is influenced not only by phenomenology but also by Zen Buddhism. See Kalmanson (2010), Sevilla (2016), and Shields (2009).

6 This interrelation is also a central theme for many phenomenologists. However, while Watsuji read both Husserl and Heidegger, his treatment of these topics emerges before and anticipates many core themes found in later thinkers like Sartre and Merleau-Ponty. And while Watsuji was deeply influenced by both Husserl and Heidegger, he was also critical of both. For example, he develops a deeply social characterization of intentionality that is, in part, a direct response to what he sees as Husserl’s excessively individualistic focus (Krueger 2020). Additionally, Watsuji develops a sustained critique of Heidegger, who Watsuji argues over-emphasizes temporality at the expense of spatiality—and as a result, fails to develop a satisfactory account of *Dasein’s* social relations. See Culbertson (2019), Johnson (2019), and Mayeda (2006).

7 Watsuji (1996, pp. 170–171).

8 (ibid., pp. 156–157).

9 (ibid., p. 157).

10 This activity can unfold both *pre-reflectively* (e.g., spontaneously touching someone’s arm mid-conversation to express intimacy) as well as in a more deliberate and *reflective* way (e.g., designing the structure of an office space to encourage regular interaction between workers).

11 Watsuji (1996, p. 157).

12 (ibid., p. 169).

13 (ibid., p. 167).

14 Watsuji occasionally uses “spatial extendedness” instead of “subjective extendedness,” which can create some interpretive confusion. For consistency, we use the latter formulation.

15 Watsuji (1996, p. 156).

16 (ibid., p. 157).

17 Krueger (2013b).

18 Within many discussions of infant-caregiver relations, there is a tendency to talk specifically about breastfeeding. We have chosen to use the word “feeding” to encompass broader practices of feeding that include both breastfeeding and bottle-feeding. We think that the dynamics of the infant-caregiver relationship here apply to both and attach no value-judgement to either practice.

19 Kaye (1982).

20 Taipale (2016).

21 Watsuji (1996, p. 62).

22 Varga and Krueger (2013).

23 Doerr-Zegers et al. (2017), Krueger and Colombetti (2018).

24 Tronick and Reck (2009).

25 Reck et al. (2004).

26 Watsuji (1996, p. 156); see also Krueger (2013a). These observations appear to anticipate what Merleau-Ponty later describes as *incorporeality* (Merleau-Ponty 2012, p. 191).

27 Watsuji, quoted in Johnson (2019, p. 135).

28 (ibid., p.135). Watsuji here again anticipates Merleau-Ponty who, some years later, writes similarly about the expressive power of the cultural world (Merleau-Ponty 2012, p. 363).

29 As Johnson (2019, pp. 135–136) observes, this confusion arises from the semantically ambiguous character of the term *ningen sonzai*, which has led some commentators to translate it as *human existence*, and others as *human being*. Watsuji uses this term in both senses, although generally at different times and in different examples. However, in some key passages, he also uses it in both senses and speaks of artifacts as potentially expressing both human existence *and* human being.

30 (ibid., pp. 135–136).

31 Watsuji (1996, p. 165).

32 (ibid., p.177).

33 Ahmed (2007).

34 Imrie and Hall (2003).

35 Krueger and Maiese (2018).

36 Krueger (2016).

37 Watsuji (1996, p. 158).

38 (ibid., p. 160).

39 (ibid., p. 160).

40 (ibid., p. 162).

41 (ibid., p. 163).

42 (ibid., p. 163).

43 (ibid., pp. 163–164).

44 (ibid., p. 164).

45 (ibid., p. 163).

46 Although see Kekki (2020), Osler (2021a, 2020a, b).

47 See Smart (2017) for an overview.

48 Smart et al. (2017, p. 11); see also Krueger and Osler (2019), Osler (2020a).

49 Watsuji (1996, p. 160).

50 Fuchs (2014).

51 (bid., p. 167).

52 (ibid., p. 168).

53 For a more detailed discussion of how we encounter the other’s lived body online, see Osler (2021a). Also see Ekdahl (forthcoming) and Ekdahl & Ravn (forthcoming) for a discussion of encountering the other via avatars in the virtual space of esports.

54 Watsuji (1996, p. 156).

55 (ibid., p. 164).

56 Again, recall Watsuji’s earlier observation that the heterogeneity of our betweenness experiences — including their affective character — covaries with the character of the artifacts and spaces that generate these experiences. This observation holds for the interactive possibilities presented by different technologies and online spaces (e.g., WhatsApp, Instagram, Facebook, Reddit, email correspondence, etc.).

57 Baym (2015), Garde-Hansen and Gorton (2013).

58 This also comes with some interesting epistemic implications. There is evidence that posts or tweets from accounts that have higher numbers of followers or friends, and that have more likes and shares, are deemed more reliable than those that garner little engagement (Smart et al. 2017).

59 Donath and Boyd (2004).

60 Note that we do not intend to bifurcate the online and offline entirely. When we go on the Internet, we are always both in online and offline space—we do not leave our bodies behind when we pick up our phones or log on to our laptops. In our contemporary world, we are frequently in “blended spaces” (Krueger and Osler 2019), where our space of activity spans both our offline and our online spheres.

61 In reality, of course, there are a number of important, practical ways that geography still impacts our Internet access. Having the infrastructure to enable Internet access is clearly necessary. However, we should also be sensitive to how governments can and do limit, monitor, and censor access to the Internet and various platforms contained therein. There is also the fact that we are bound by linguistic limitations. While you can enter sites that are written in Finnish, one’s ability to engage on those platforms is curtailed if one cannot actually speak, read, or write Finnish.

62 Watsuji (1996, p. 163).

63 (ibid., p. 162).

64 For a discussion of the kinds of community that we find online, see Osler (2020a).

65 For an excellent in-depth analysis of epistemic bubbles and echo chambers in the online sphere, see Nguyen (2020).

66 Watsuji (1996, p. 160).

67 (ibid., p. 155).

68 Pariser (2011), Nguyen (2020).

69 These are just some of the many political and ethical issues surrounding online space. Watsuji was a deeply ethical thinker; as we noted at the start, his phenomenological analysis is always developed in the service of his ethics. So, in addition to exploring experiential dimensions of online space in the way we have done here, he would insist that we also remain mindful of the political and ethical issues (i.e., concerning access, power, transparency, and control) we briefly touched upon above—as well as other issues we have not addressed. Despite its immense power and potential to bring people together and forge new forms of betweenness, the Internet is not, after all, an unqualified good.

## References

[CR1] Ahmed, Sara. 2007. A phenomenology of whiteness. *Feminist Theory* 8. SAGE Publications: 149–168.

[CR2] Baym Nancy K (2015). Personal connections in the digital age.

[CR3] Culbertson, Carolyn. 2019. The genuine possibility of being-with: Watsuji, Heidegger, and the primacy of betweenness. *Comparative and Continental Philosophy* 11. Routledge: 7–18.

[CR4] Doerr-Zegers Otto, Irarrázaval Leonor, Mundt Adrian, Palette Virginie (2017). Disturbances of embodiment as core phenomena of depression in clinical practice. Psychopathology.

[CR5] Donath Judith, Boyd Danah (2004). Public displays of connection. BT Technology Journal.

[CR6] Ekdahl, David. forthcoming. Mechanical keyboards and crystal arrows: Incorporation in esports. *Journal of Consciousness Studies*.

[CR7] Ekdahl, David, and Susanne Ravn. forthcoming. Social bodies in virtual worlds: Intercorporeality in esports. *Phenomenology and the Cognitive Sciences*. 10.1007/s11097-021-09734-1 PMC8075606 33935609

[CR8] Fuchs Thomas (2014). The virtual other: Empathy in the age of virtuality. Historical Journal of Film, Radio and Television.

[CR9] Garde-Hansen Joanne, Gorton K (2013). Emotion online: Theorizing affect on the internet.

[CR10] Imrie Rob, Hall Peter (2003). Inclusive design: Designing and developing accessible environments.

[CR11] Johnson, David W. 2019. *Watsuji on nature*. Northwestern University Press.

[CR12] Kalmanson Leah (2010). Levinas in Japan: The ethics of alterity and the philosophy of no-self. Continental Philosophy Review.

[CR13] Kaye Kenneth (1982). The mental and social life of babies: How parents create persons.

[CR14] Kekki Minna-Kerttu (2020). Authentic encountering of others and learning through media-based public discussion: A phenomenological analysis. Journal of Philosophy of Education.

[CR15] Krueger, Joel. 2013a. Watsuji’s phenomenology of embodiment and social space. *Philosophy East & West* 63. University of Hawai’i Press: 127–152.

[CR16] Krueger Joel (2013). Ontogenesis of the socially extended mind. Cognitive Systems Research.

[CR17] Krueger, Joel. 2016. The extended mind and religious cognition. In *Religion: Mental religion. Part of the Macmillan Interdisciplinary Handbooks: Religion series*, ed. Niki Kasumi Clements, 237–254. MacMillan.

[CR18] Krueger Joel, Taguchi Shigeru, Altobrando Andrea (2019). Watsuji’s phenomenology of aidagara: An interpretation and application to psychopathology. Tetsugaku companion to phenomenology and Japanese philosophy.

[CR19] Krueger Joel (2020). Watsuji, intentionality, and psychopathology. Philosophy East & West.

[CR20] Krueger Joel, Colombetti Giovanna (2018). Affective affordances and psychopathology. Discipline Filosofiche.

[CR21] Krueger Joel, Maiese Michele (2018). Mental institutions, habits of mind, and an extended approach to autism. Thaumàzein.

[CR22] Krueger Joel, Osler Lucy (2019). Engineering affect: Emotion regulation and the techno-social niche. Philosophical Topics.

[CR23] Mayeda Graham (2006). Time, space and ethics in the philosophy of WatsujiTetsurō, Kuki Shuzo, and Martin Heidegger.

[CR24] McCarthy, Erin. 2011a. Beyond the binary: Watsuji Testurō and Luce Irigaray on body, self, and ethics. In *Japanese and continental philosophy: Conversations with the Kyoto School*, ed. Bret Wingfield Davis, Brian Schroeder, and Jason Wirth, 212–228. Bloomington: Indiana University Press.

[CR25] McCarthy Erin (2011). Ethics embodied: Rethinking selfhood through continental, Japanese, and feminist philosophies.

[CR26] Merleau-Ponty, Maurice. 2012. *Phenomenology of perception*, trans. Donald Landes. New York: Routledge.

[CR27] Nguyen CT (2020). Echo chambers and epistemic bubbles. Episteme.

[CR28] Osler Lucy (2020). Feeling togetherness online: a phenomenological sketch of online communal experiences. Phenomenology and the Cognitive Sciences.

[CR29] Osler Lucy (2020). See you online. The Philosophers’ Magazine.

[CR30] Osler, Lucy. 2021a. Taking empathy online. *Inquiry: An Interdisciplinary Journal of Philosophy*. 10.1080/0020174X.2021.1899045.

[CR41] Osler, Lucy. 2021b. Interpersonal atmospheres: an empathetic account. Doctoral thesis, University of Exeter.

[CR31] Pariser Eli (2011). The filter bubble: How the new personalized web is changing what we read and how we think.

[CR32] Reck Corinna, Hunt Aoife, Fuchs Thomas, Weiss Robert, Noon Andrea, Moehler Eva, Downing George, Tronick Edward Z, Mundt Christoph (2004). Interactive regulation of affect in postpartum depressed mothers and their infants: An overview. Psychopathology.

[CR33] Sevilla Anton Luis (2016). The Buddhist roots of WatsujiTetsurō’s ethics of emptiness. The Journal of religious ethics.

[CR34] Shields, James M. 2009. The art of aidagara: Ethics, aesthetics, and the quest for an ontology of social existence in Watsuji Tetsurō’s Rinrigaku. *Asian Philosophy* 19. Routledge: 265–283.

[CR35] Smart Paul (2017). Extended cognition and the internet. Philosophy & Technology.

[CR36] Smart Paul, Heersmink Richard, Clowes Robert W, Cowley Stephen J, Vallée-Tourangeau Frédéric (2017). The cognitive ecology of the internet. Cognition beyond the brain: Computation, interactivity and human artifice.

[CR37] Taipale Joona (2016). Self-regulation and beyond: Affect regulation and the infant-caregiver dyad. Frontiers in Psychology.

[CR38] Tronick Edward Z, Reck Corrina (2009). Infants of depressed mothers. Harvard Review of Psychiatry.

[CR39] Varga Somogy, Krueger Joel (2013). Background emotions, proximity and distributed emotion regulation. Review of Philosophy and Psychology.

[CR40] Watsuji, Tetsurō. 1996. *Watsuji Tetsuro’s Rinrigaku: Ethics in Japan*. Trans. Seisaku Yamamoto and Robert Edgar Carter. Albany: SUNY Press.

